# Ruminative minds, wandering minds: Effects of rumination and mind wandering on lexical associations, pitch imitation and eye behaviour

**DOI:** 10.1371/journal.pone.0207578

**Published:** 2018-11-19

**Authors:** Mariana Rachel Dias da Silva, Dorottya Rusz, Marie Postma-Nilsenová

**Affiliations:** Tilburg University Cognitive Science and Artificial Intelligence Department, Warandelaan 2, 5037AB, Tilburg, The Netherlands; Radboudumc, NETHERLANDS

## Abstract

This study demonstrates that rumination is reflected in two behavioural signals that both play an important role in face-to-face interactions and provides evidence for the negative impact of rumination on social cognition. Sixty-one students were randomly assigned either to a condition in which rumination was induced or to a control condition. Their task was to play a speech-based word association game with an Embodied Conversational Agent during which their word associations, pitch imitation and eye movements were measured. Two questionnaires assessed their ruminative tendencies and mind wandering thoughts, respectively. Rumination predicted differences in task-related mind wandering, polarity of lexical associations, pitch imitation, and blinks while mind wandering predicted differences in saccades. This outcome may show that rumination has a negative impact on certain aspects of social interactions.

## Introduction

We all experience our thoughts drifting away while attempting to concentrate on a task, whether it be reading an article, listening to a lecture, writing a paper, or even having a conversation. Indeed, such mind wandering (MW) thoughts comprise up to half of our daily thoughts [1, 2]. MW has been defined as self-generated thought which is active and independent of perceptual input, often unrelated to the task at hand and directed to goals that extend beyond the here and now [3–6]. However, in some cases, MW may also be task-related (e.g.:“I wonder how long it will take me to finish reading this article.”) [6, 7]. The past decade has seen a substantial increase in the understanding of how MW thoughts emerge and the reasons for their occurrence. Their costs include decreased text comprehension [8, 9], higher variability in reaction times [10], increased number of errors in both memory, working memory [11] and choice reaction time tasks [7, 12], as well as lower measures in general aptitude [11] and increases in negative mood [2]. Indeed, MW can have negative consequences; however, it also provides freedom from immediacy and has been associated with creativity in unusual uses tasks [13] and future planning [6, 14, 15]. Given its pervasiveness in our lives, it may reflect an essential adaptation of the mind, serving to maintain a coherent sense of self by integrating the past and present self with future experiences [15–18]. MW has been studied in the laboratory during various interactive tasks as well as in daily-life; however, most of these studies have not focused on the social nature of our mind wandering thoughts and their role in our daily interactions [19]. The present study aims to fill this gap in research on MW by investigating social and cognitive cues to ruminative and MW episodes and exploring the implications for social interactions.

### Personal goals, negative mood, and mind wandering

MW thoughts are often directed towards personal goals that are not directly related to the task at hand [14], but are associated with current life concerns [4], which suggests that the adaptive function of MW can facilitate problem-solving in daily life [5, 20]. Within the context of personal goals, a negative mood may indicate personal problems [21] that individuals may try to solve during MW. Both the induction of personal concerns [17, 22, 23], as well as induction of negative moods have been shown to increase MW [24].

When MW about problems and concerns leads to effective problem solving, this may have positive consequences on both cognition and affect. However, when ineffective, MW heightens the salience of the current problem or concern, which can in turn have negative affective and cognitive consequences [20]. Studies indicate that it is unclear whether negative mood precedes MW [6, 20, 24, 25], yet it has been proposed that negative mood influences the affective content of MW, making mood congruent thoughts more accessible [20, 26]. The type of MW hence depends on the format and content of MW thoughts, which dictates whether the thoughts will have (mal)adaptive outcomes.

### Rumination and wandering

Rumination is characterised in terms of persistent and recurring self-reflective thoughts about a particular theme that deviate attention away from relevant themes and current tasks in the immediate external environment [27, 28]. Whenever MW becomes rigid and inflexible in the form of ruminative, perseverative cognition, it may become a risk factor [17, 29]. That is, when MW loses its expansive, adaptive form, it may under certain circumstances lock into a repetitive spiral of homogeneous negatively-valenced thoughts [30]. The first objective of the current study is to corroborate previous research [31, 32] by examining if rumination increases frequencies of MW. On the one hand, MW and rumination may be seen as antithetical concepts, where MW is seen as a form of free, unguided, internal thought, while rumination is thought that is fixed around a single theme [3, 33]. On the other hand, rumination may be seen as a style of thinking which may take hold of a wandering thought, lock it into a spiral of repetitive self reflection, and impede individuals from focusing on the task at hand [30, 34–37]. As MW and rumination are inevitably linked [17], this study aims to integrate these concepts, motivated by the fact that one cannot discuss rumination without taking into consideration that it is a style of MW. Nor can one discuss MW without taking into account the possibility that a particular portion of these self-generated thoughts may fall captive to a ruminative style of thinking. Although the majority of research presupposes MW to refer to task-unrelated thoughts alone, other methodological perspectives deviate from such a view [6, 7, 32, 38] and consider there to be two types of self-generated thought, namely, interfering thoughts concerning appraisal of one’s performance on a task (task-related interference, TRI), and thoughts directed towards information that is unrelated to the current environment or to the current task (task-unrelated thinking, TUT) [38]. The rigid and inflexible quality of ruminative thought appears to be closely related to the rigid characteristics of task-related interferences. [31, 32]. At the same time, rumination has the potential to exacerbate the relationship between pre-existing dyspohoria and increases in task-unrelated thoughts. [32].

Rumination can be divided into two major subcategories, namely, reflective pondering and brooding [39]. Self-reflection suggests a purposeful inward focus aimed at cognitive problem solving. Brooding, on the other hand, involves a comparison of one’s current situation with some unachieved standard. Often, brooding is associated with a decreased controllability of thoughts. Rumination can be adaptive when self-reflection serves to solve problems, however, it may become maladaptive when either reflection or brooding lead an individual passively think about their problems and feel helpless in finding solutions. Such maladaptive rumination has been found to be associated with the excessive elaboration of negative information [40]. Moreover, high trait rumination has been associated with enhanced recollection memory for negative words in young females, also after controlling for negative mood [41]. Hence, the second objective of this study is to expand upon previous research and investigate whether rumination is related to the production of more negative lexical associations in a task.

### Ruminative self-focus in social cognitive mechanisms

A significant proportion of MW thoughts concerns others [16, 42]; i.e., it is interpersonally-focused and social in nature [19]. Various studies have investigated MW in interactive settings, such as in the classroom, both online [43] and offline [44], as well as with intelligent tutoring systems [45, 46]. Although most of this research is social and interactive by nature, it has primarily investigated performance-related consequences of mind wandering in these interactive settings. However, the social and relational consequences of the mind wandering have been largely neglected. The current study addresses this gap in the literature and highlights the need to investigate behavioural social cues to MW in interactive environments. Based on past research, we hypothesise that MW thoughts might be detectable through social cognitive mechanisms such as pitch imitation and eye movements, which provide valuable information about individuals’ emotions, mental states, and behaviours during social interactions [47, 48].

#### Pitch imitation

Speakers have frequently been shown to accommodate to one another’s pitch patterns, as pitch is perhaps the most important indicator of the emotions and attitudes of a speaker [49, 50]. The ability to correctly perceive pitch in another’s speech and to adapt one’s pitch according to one’s goals is an essential communicative and social skill (Communication Accommodation Theory; [51–55]). Pitch convergence may then be an indicator of awareness towards one’s environment and of desire for social approval and acceptance [56–59].

Neuroimaging evidence suggests that reduced imitation is associated with self-related processing and independent self-construals [60]. Moreover, experimentally induced self-focus inhibits imitation [61, 62]. Additionally, depressed individuals, who tend to have more negative thoughts, have also been found to show less behavioural imitation [63], while dysphoric and depressed individuals have failed to express normal facial imitation of both positive and negative facial expressions [64]. Consolidating the association between self-focus and reduced imitation, the third objective of this study is to investigate whether rumination, characterised by recurring, self-focused thoughts, is associated with reduced (pitch) imitation.

#### Gaze behaviour

An important cue signalling joint attention between interaction partners is gaze behaviour [65]. It provides important information about people’s social and cognitive behaviours [48, 66] and is indicative of visual attention processes [67, 68]. Only few studies have investigated eye movements in relation to rumination [69, 70]; and various studies have investigated eye movements and MW [68] in a range of tasks including reading, driving, tasks of sustained attention, and learning with an intelligent tutoring system [67, 71–75].

During social interactions, eye contact serves as a signal of joint attention and interest between interaction partners [65]. In MW studies, fixations and saccades decrease and average fixation duration becomes longer [67, 72, 73], indicating that eye movements are both slower and less frequent during MW episodes, which may be indicative of increased cognitive inflexibility [17]. In a study by Rauthmann and colleagues [48], individuals who scored high on neuroticism, a personality trait highly tied to rumination, had less and longer fixations and spent more time dwelling on an abstract animation. With respect to blinks, Smilek and colleagues [72] found that participants blinked more during MW than during on-task episodes. More blinks have also been shown to indicate the exchange of attention from the external task at hand to internal thoughts, and were thus associated with reduced attention and increased error in the processing of external information [72, 76]. In sum, less active saccades, less and shorter fixations, and more blinks may represent reduced attention towards the external environment during MW. The third objective of this study is then to build upon and complement previous literature on MW and eye movements by investigating to what extent rumination affects eye movements in an interactive setting.

### Current study

The present study was designed as a speech-based word association game with an Embodied Conversational Agent in order to investigate how rumination affects participants’ lexical associations. A word association game with an Embodied Conversational Agent was used in this study because of its relative simplicity, enabling us to control for any noise that might arise from more ecologically valid contexts, such as a free conversation with an interaction partner. At the same time, it is less constrained than a simple speech shadowing task in a non-interactive setting [54, 55, 77], in which participants are limited to only repeating isolated words [78]. In contrast, participants have the freedom to come up with their own word associations in this game. This study is innovative in that it explores two social and cognitive mechanisms, namely, pitch imitation and eye behaviour as possible behavioural cues to rumination and mind wandering, and addresses their implications during social interactions. The guiding question in this research then is: To what extent does self-focused rumination affect MW, negative lexical associations, pitch imitation and eye behaviour? We propose the following hypotheses: H1) Rumination will result in increases in MW; H2) Rumination will increase the occurrence of negative lexical associations; H3) Rumination will negatively affect imitation, and finally; H4) Rumination will affect eye movement behaviours; more specifically, it will be associated with more blinks, less saccades, less fixations, and longer average fixation duration.

## Methods

### Participants and design

**Fig 1:**
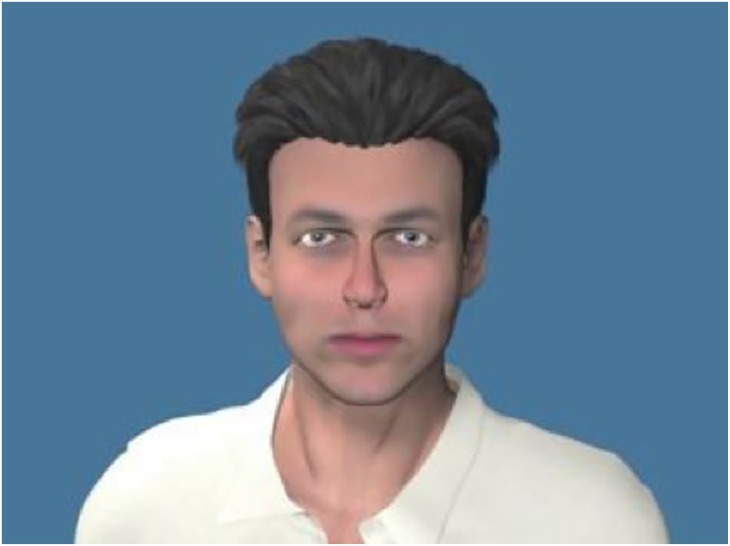
Embodied Conversational Agent.

Sixty-two English speaking students were recruited from the student population at Tilburg University. Participants were aged 18 to 33 years; mean age was 23.82 years (*SD* = 2.50). This sample size was sufficient for detecting the smallest effect size of interest (SESOI) according to Simonsohn (2015)’s [79] recommendation to set the smallest effect size of interest so that the original experiment had 33% power to reject the null hypothesis if this effect size was true. The SESOI for this experiment was based on Smallwood and O’Connor (2005) [32] for MW, Mattheij and collegues (2015) for pitch, and lastly, Smilek and colleagues (2010) and Rauthmann and colleauges (2012) for eye movements [31, 32, 48, 49, 72]. Thirty-one males and 31 females participated, but data from one male participant had to be excluded due to a procedural error. The study was approved by the Tilburg University Institutional Review Board, and written informed consent was obtained from each participant at the beginning of the experimental session. Participants took part in an interactive task presented as a word association game with an Embodied Conversational Agent (Fig 1). The between-participant manipulation was induced state rumination. The within-participant factor in the task was the Embodied Conversational Agent’s vocal pitch (High/Low). The dependent variables measured were self-reported MW, the polarity of the words that participants generated during the word association game (positive, negative, neutral), pitch imitation parameters, and eye movement parameters (blinks, saccades, fixations, and average fixation duration).

### Materials and instrumentation

#### Trait rumination

We used the Rumination Inventory (S1 File, [80]), which was designed to measure a tendency toward distractability, a tendency to engage in mental rehearsal of future and past events, and a tendency toward repetitive thought, including increased frequency and decreased controllability of thoughts [81]. Participants had to indicate whether the statements (e.g., “I often get distracted from what I am doing with thoughts about something else” and “I have no trouble focusing all of my attention on one thing”) describe them well or not on a scale from 1-7. The scale contained 10 items and was found to be reliable (*α* = .67). Participants were asked to complete the RI for a second time at the end of the experiment (*α* = .75).

#### State rumination

In the rumination induction (S2 File) procedure, treated as a proxy for state rumination, participants were asked to think about a variety of (45) recurring self, emotion, and symptom focused thoughts (e.g., “your character and who you strive to be” and “the way you feel inside”). In the control condition, participants thought about 45 items that were not related to the self, emotions, or symptoms (e.g., “the shape of the continent of Africa” and “a group of polar bears fishing in a stream”; [82]).

#### Word association task

The word association task consisted of twenty-two trials, in which an Embodied Conversational Agent produced either a high or low pitch voice when uttering monosyllabic words in a word association game. The participant was instructed to react within four seconds with the first verbal association that came to mind. A word association game in which a user plays with an Embodied Conversational Agent was chosen as a task because it was found to be engaging and interactive, while allowing for a controlled design. The experimental setup consisted of a desktop computer that ran the experimental software (E-prime), a headset (MB Quart—K800) and an Eye Tracker (SMI Red 250). Participants were placed approximately 70 centimeters in front of the screen. The software E-Prime was used to run the experiment. Stimulus material was presented with a display refresh rate of 60 Hz on a #4C759C color (light blue) background by an Embodied Conversational Agent who uttered monosyllabic synthesised words with the same facial expression. The Embodied Conversational Agent was created by Postma-Nilsenová and colleagues [50] with the software Poser (Smith Micro Software Inc, Aliso Viejo, California, USA). Stimulus material was composed of five-second long .avi files, in which the Embodied Conversational Agent uttered one word after which the participant had four seconds to respond. Lexical properties: The twenty-two words (S3 File) used in the word association task were related to the academic, university context. As all participants were students from Tilburg University, we used semantically neutral words that they encounter in their university day-to-day lives (e.g. teach, time, add, goal, class, etc.). The words were synthesised using a publicly available commercial software and recorded with Audacity 2.0.0. The audio recordings were edited with Praat 5.3.04 [83] and rescaled so that all had an intensity of 70 decibels. Eighty milliseconds were added before each utterance, and subsequently were resynthesised with an LPC resynthesiser in Praat. In order to make the variation in pitch perceptually distinct, the high and low-pitch stimuli differed by 40 Hz on average, where half of the recorded stimuli were resynthesised 20 Hz higher, and the other half, 20 Hz lower than the original recording (S3 File).

#### Mind wandering

In order to measure MW, we used a subjective measure of task-unrelated thought (S4 File, Thinking component of the Dundee Stress Questionnaire; [38]). This scale assesses what participants are thinking about during a recently completed task. It contains two 8-item parts: (1) one measuring task-related interferences (TRI; e.g., “I thought about how I should work more carefully”) and (2) one referring to task-unrelated thoughts (TUT; e.g., “I thought about personal worries”). Participants were asked to indicate on a 5-point Likert scale how well each of the statements described them, ranging from 1(never) to 5 (very often). The scale contained 16 items and was found to be highly reliable (*α* = .89). Reliability coefficients for each subscale are reported in Table 1.

**Table 1:** Descriptive statistics for rumination, MW (TRI/TUT), eye movements, pitch, and lexical associations (N = 61).

	**Rumination** *N* = 30			**Control** *N* = 31				
	*M*	*Mdn*	*SD*	*M*	*Mdn*	*SD*	*α*	*p*
**Tests**								
Trait Rumination T1^a^	5.07		0.70	4.72		0.79	.66	.074
Trait Rumination T2^b^	5.05		0.84	4.57		0.85	.75	.030
MW	2.39		0.76	1.98		0.61	.89	.024
TRI	2.84		0.84	2.39		0.73	.79	.029
TUT	1.93		0.85	1.56		0.65	.87	.062
**Pitch**^c^								
Low Pitch	6.22		4.95	5.57		4.55		.603
High Pitch	6.19		5.20	6.38		4.78		.878
Lexical Associations*								
Positivity (SWN)	0.07	0.05	0.05	0.08	0.07	0.04		.231
Objectivity (SWN)	0.76	0.78	0.12	0.80	0.81	0.12		.173
Negativity (SWN)	0.20	0.08	0.62	0.06	0.05	0.05		.020
Positive (LIWC)	5.28	1.35	8.35	5.20	4.55	6.00		.663
Negative (LIWC)	10.86	9.31	8.88	6.96	5.26	6.93		.052
**Eye Movements** *N* = 59	**Rumination** *N* = 29			**Control** *N* = 30				
Blinks*	2.87	2.32	2.27	2.77	2.50	1.55		.485
Saccades	11.59		5.72	11.02		5.38		.696
Fixations	10.08		4.07	10.57		4.07		.649
Av.Fix. Dur.^d^	659.49		367.59	617.91		252.4		.616

^a^ Overall Mean for the first Rumination Inventory was (*M* = 4.57, *SD* = 0.85)

^b^ Rumination reported at the end of the experiment.

^c^ Pitch is reported in semitones.

^d^ Average fixation duration is reported in milliseconds.

* Variables with non-normal distributions; p-values derived from Mann-Whitney tests.

### Procedure

Participants arrived in the lab and were asked to sit in the sound-proof booth in front of a computer screen where they first filled out the Rumination Inventory [80]. In order to ensure that participants in both conditions were in a comparable mood, participants watched a ninety second long relaxation video consisting of underwater scenes and accompanied by soothing music (S5 File, [84]). Then they were randomly assigned to either the rumination induction or to the control group. After the rumination/ control task, they played a word association game with the Embodied Conversational Agent in which their voice and eye movements were recorded. Participants followed the instructions on the screen and were asked to sit as still as possible in order for the device to capture their eye movements accurately. Before having their eye movements recorded, they underwent a calibration and validation procedure in order to ensure that the eye tracker was measuring correctly. Next, the Embodied Conversational Agent began the word association task, pronouncing either high- or low-pitched words. Each stimulus was preceded by a centralised fixation cross that was on the screen for one second. Participants then had to pronounce the first association that came to mind, within four seconds. After the word association task, participants had to fill out two post-questionnaires; one about their MW episodes during the task (Thought Component of the Dundee Stress State Questionnaire (DSSQ) and the Rumination Inventory for the second time, in order to verify the effectiveness of the rumination induction. A full version of the materials is available in the supplementary files.

### Pitch data analysis

The aim of the auditory data analysis was to determine whether there was a significant change in the vocal pitch of participants in response to a low or high-pitched utterance by the Embodied Conversational Agent, and whether this change was higher for participants in the rumination condition compared to the control. The recordings were analysed with Praat 5.3.04. They were first visually inspected in order to establish a pitch floor and ceiling for the speakers to prevent errors. As a result, the range was set to 40 Hz—400 Hz for both the male and female voices. Prior to the analysis of the word recordings, extraneous noises and non-speech sounds (pauses, hesitations, clearing of the throat, and background noises) were edited from the recordings and octave jumps were manually corrected. Creaky voices and octave jumps frequently result in errors of pitch determination, and hence had to be manually corrected [85–87]. This required visual and auditory inspection of each of the 1233 recordings in order to remove pauses, hesitations, stuttering, creaky voices and octave jumps. The mean pitch for each segment was determined with the standard autocorrelation-based pitch detection in Praat in semitones [83]. A total of 1233 pitch measurements were obtained for 1342 (22 × 61) of the experimental trials. Output was missing for 109 of the trials (8.12%) where participants were unable to come up with a word in the association task within the allotted time. Pitch measurements were averaged per participant and per condition (high or low pitch uttered by the Embodied Conversational Agent).

### Lexical data analysis

Prior to the lexical analysis several words had to be adjusted in order to be recognised by the semantic analysis tools. Words in the past tense had to be modified to present tense and plural words had to be changed to their singular form. When the response of the participants contained more than one word (e.g., “hard work”), the words were analysed separately, and both words were included in the final analysis. When the participant was undecided about an answer and uttered multiple words, the first word was always analysed (e.g., “words/limit/word limit”).

The polarity of the words (positive/negative) was analysed with LIWC (Linguistic Inquiry and Word Count, [88]), a text analysis software that is widely used in a broad range of experimental settings to evaluate emotionality [89]. Since LIWC may not have always captured the meaning of compounds (e.g., ‘give up’ would be coded as two separate items with positive polarity, while the expression as a whole has negative polarity), the lexical associations were also analysed by SentiWordNet [90], a publicly available lexical tool for opinion mining. SentiWordNet determines the polarity of a word by assigning three numbers to it, a positivity, a negativity and an objectivity value that always add up to one [90]. In SentiWordNet, contrary to the LIWC, compound words are recognised as single entries.

### Eye movement data analysis

Participants’ eye movements were recorded with an SMI RED 250 eye-tracking device, with a sampling rate of 250 Hz positioned below a Dell computer (22-inch monitor, 1680x1050 resolution). This system uses infrared tracking technology which measures pupil center and size of both eyes. Blinks, saccades, fixations, and average fixation duration were processed in MATLAB R2015a (8.5.0) in order to prepare the data for statistical testing. Often in eye tracking experiments measuring mind wandering, if the quality of data is low (e.g. due to loss of signal or a participant not facing the eye tracker), data points or entire participants are excluded from the analysis [45, 74]. Following such practices, eye movement data for 2 participants were excluded, as there were insufficient eye movements recorded for over 50% of these participants’ trials.

## Results

Data were analysed for 61 participants. Means, Standard Deviations, and Cronbach’s *α*’s are displayed in Table 1. Pitch, lexical associations and eye movements were averaged across trials and aggregated to the participant level. We first performed a Kolmogorov-Smirnov test of normality for rumination measured at the beginning and end of the experiment, MW(TRI/TUT), high and low pitch, each eye movement parameter, and lexical associations in order to evaluate the distribution of values in comparison to the standard normal distribution. Rumination measured prior to the experiment, MW, saccades, and fixations were normally distributed. For blinks, lexical parameters and pitch, the test indicated a non-normal distribution of values (*p* < 0.001). A subsequent visual inspection did not reveal an inordinate amount of violations against the normality distribution for pitch, with only a slight curving away of points from the q-q plot line; hence the assumption of normality appeared to be reasonable for this variable. Blinks and lexical parameters were analysed with the help of non-parametric tests wherever applicable.

### Effect of rumination

A mixed ANOVA with the first and second measures of rumination as within-participant variables and condition (rumination/control) as the between-participant variable, indicated that there was a significant effect of the experimental manipulation on participants’ rumination tendencies, with main effects found for condition *F*(1, 59) = 4.50, *p* = .038, $\eta_{p}^{2}=.07$. No main effects were found for trait rumination, *F*(1, 59) = 2.21, *p* = .143, $\eta_{p}^{2}=.04$ and there were no interaction effects between trait rumination and condition *F*(1, 59) = 1.20, *p* = .278, $\eta_{p}^{2}=.02$. Means and standard deviations (Table 1) reveal that participants in both the rumination induction condition and in the control group were already highly ruminative prior to the manipulation. There were no significant differences between both groups at T1. However, at T2, means for trait rumination in the control group were significantly lower than means for the rumination group, which on average, stayed the same from T1 to T2. Although we expected the rumination induction to have led to a significant increase in rumination scores, the reverse occurred, and instead, there was a significant decrease in rumination scores in the control group. The fact that the rumination induction did not lead to an increase in rumination scores was a caveat in this study; therefore, to pry out the effects of the rumination manipulation from trait rumination, we introduced trait rumination as a control variable and treated induced rumination as a proxy for state rumination for all subsequent analyses.

#### Mind wandering

In order to test whether rumination increased MW, a two-way ANOVA was performed on answers to the Thinking Component of the Dundee Stress Questionnaire with both trait rumination and state rumination as covariates. There was a marginally statistically significant effect of both trait rumination *F*(1, 58) = 3.54, *p* = .065, $\eta_{p}^{2}=.06$ and state rumination *F*(1, 58) = 3.22, *p* = .078. $\eta_{p}^{2}=.05$ on TRI scores, but no effect of either trait rumination *F*(1, 58) = 4, *p* = .409, $\eta_{p}^{2}=.01$ or state rumination *F*(1, 58) =, *p* = .102, $\eta_{p}^{2}=.05$ on TUT scores.

#### Lexical associations

As the negative associations were not normally distributed, a Mann-Whitney test was performed in order to test if there was a significant effect of state rumination on participants’ generation of negative lexical associations. Descriptive statistics are shown in Table 1. When comparing the scores provided by the LIWC, the results of the test indicated that participants came up with marginally significant more negative words in the rumination (*Mdn* = 9.31) than in the control condition (*Mdn* = 5.26), *U* = 331.00, *z* = −1.94, *p* = .052, while the negativity scores from SentiWordNet were significantly higher in the rumination condition (*Mdn* = 0.08) than in the control condition (*Mdn* = .05), *U* = 303.50, *z* = −2.33, *p* = .020. As there were no nonparametric alternatives which would also account for the effect of trait rumination on lexical associations, we also performed a two-way ANOVA with state and trait rumination as covariates. There was no effect of either state rumination *F*(1, 58) = 2.40, *p* = .127, $\eta_{p}^{2}=.04$, or trait rumination on the LIWC scores *F*(1, 58) = 2.11, *p* = .152, $\eta_{p}^{2}=.04$. Moreover, there was no effect of either state rumination *F*(1, 58) = 1.72, *p* = .195, $\eta_{p}^{2}=.029$, or of trait rumination on the SentiWordNet scores, *F*(1, 58) = 0.15, *p* = .700, $\eta_{p}^{2}<.01$.

#### Pitch imitation

In order to test whether there was a significant effect of rumination on pitch accommodation, a mixed ANOVA was performed. The within-participant factors were high and low pitch, while the between-participant factor was state rumination, with trait rumination as a covariate. Pitch imitation was operationalised as adaptation to the Embodied Conversational Agent’s pitch, so that high-pitched utterances would have induced higher pitch responses, while lower-pitched utterances would have induced lower pitch responses. There was a statistically significant interaction between state rumination and high and low pitch, *F*(1, 58) = 8.23, *p* = .006, $\eta_{p}^{2}=.12$, as well as between trait rumination and high and low pitch, *F*(1, 58) = 5.22, *p* = .026, $\eta_{p}^{2}=.08$. In the control condition, mean pitch was lower following vocalizations by a low-pitch prime (*M* = 5.57 semitones, *SD* = 4.55 semitones), and mean pitch was higher following vocalizations by a high pitch prime (*M* = 6.38 semitones, *SD* = 4.78 semitones). In the rumination condition, this was not the case, as there were no significant differences in pitch after either a low prime (*M* = 6.22 semitones, *SD* = 4.95 semitones) or a high prime (*M* = 6.19 semitones, *SD* = 5.20 semitones). Follow-up comparisons indicate a significant effect (*d* = 0.50) of the high and low pitch experimental manipulation in the control condition (*M* = 0.81, *SD* = 1.61), *t*(30) = 2.79, *p* = .009, 95% CI [0.22, 1.40], indicating that participants in the control condition accommodated their pitch significantly more to the Embodied Conversational Agent’s pitch, while those in a ruminative state did not (*M* = 0.03, *SD* = 1.15), *t*(29) = 0.14, *p* = .891, 95% CI [-0.45, 0.40].

### Rumination and mind wandering as predictive of eye movements

Separate regressions were conducted in order to verify the effect of state and trait rumination, as well as mind wandering on eye movements. In this analysis, we included mind wandering as combination of the TUT and TRI scales a predictor, as our hypotheses for eye movements derived from studies investigating either rumination or mind wandering in general in relation to eye movements. The regression model in Step 1 predicted the number of blinks when MW and the condition were not included in the analysis. The results of the regression indicated that trait rumination explained 7% of the variance in the number of blinks, *R* = .27, adjusted-*R*^2^ = .05, Δ*R*^2^ = .07, *F*(1, 57) = 4.32, *p* = .042. Regression coefficients of the predictors of the number of blinks are shown in Table 2.

**Table 2:** Predictors of the number of blinks.

	*B*	*SE(b)*	*β*	t	*p*
**Step1**					
(Constant)	-1.72	2.20		-0.78	.437
Trait Rumination	0.97	0.47	0.27	2.08	.042*
**Step2**					
(Constant)	-1.79	2.22		-0.81	.424
Trait Rumination	0.91	0.49	0.25	1.86	.068
MW	0.16	0.36	0.06	0.43	.672
**Step 3**					
(Constant)	-2.64	2.72		-0.97	.337
Trait Rumination	0.98	0.51	0.27	1.93	.059
MW	0.19	.37	0.07	0.52	.605
State Rumination	0.29	0.53	0.08	0.55	.588

Note: *R*^2^ = .07 for Step 1; Δ*R*^2^ = .00 for Step 2; Δ*R*^2^ = .00 for Step 3.

Note: *F* = 4.32* for Step 1; Δ*F* = .18 for Step 2; Δ*F* = .30 for Step 3.

* Δ*F* is significant at the 0.05 level (2-tailed).

The regression model in Step 2 explained 11.0% of the variance in the number of saccades, *R* = .33, adjusted-*R*^2^ = .08, Δ*R*^2^ = .08, *F*(2, 56) = 3.41, *p* = .040 and significantly predicted the number of saccades when MW and trait rumination were also included in the model. Regression coefficients of the predictors of the number of saccades are shown in Table 3.

**Table 3:** Predictors of the number of saccades.

	*B*	*SE(b)*	*β*	t	*p*
**Step1**					
(Constant)	2.12	6.43		0.33	.743
Trait Rumination	1.96	1.37	0.19	1.44	.156
**Step2**					
(Constant)	1.16	6.25		0.19	.853
Trait Rumination	1.14	1.38	.11	0.83	.413
MW	2.20	1.02	0.28	2.15	.036*
**Step3**					
(Constant)	-0.89	7.68		-0.12	.909
Trait Rumination	1.31	1.44	0.13	0.91	.366
MW	2.30	1.05	0.29	2.18	.033
State Rumination	0.69	1.49	0.06	0.47	.643

Note: *R*^2^ = .04 for Step 1; Δ*R*^2^ = .08 for Step 2; Δ*R*^2^ = .00 for Step 3.

Note: *F* = 2.06 for Step 1; Δ*F* = 4.62* for Step 2; Δ*F* = .22 for Step 3.

*Δ*F* is significant at the 0.05 level (2-tailed).

Neither trait rumination, state rumination, nor mind wandering was a significant predictor variable of the number of number of fixations (Table 4).

**Table 4:** Predictors of the number of fixations.

	*B*	*SE(b)*	*β*	t	*p*
**Step1**					
(Constant)	4.91	4.75		1.04	.305
Trait Rumination	1.16	1.01	0.15	1.15	.255
**Step2**					
(Constant)	4.60	4.76		0.97	.339
Trait Rumination	0.89	1.05	0.12	0.85	.402
MW	0.72	0.78	0.13	0.92	.360
**Step3**					
(Constant)	1.09	5.81		0.19	.852
Trait Rumination	1.18	1.09	0.15	1.09	.282
MW	0.88	0.80	0.15	1.11	.272
State Rumination	1.19	1.13	0.15	1.06	.296

Note: *R*^2^ = .02 for Step 1; Δ*R*^2^ = .02 for Step 2; Δ*R*^2^ = .02 for Step 3.

Note: *F* = 1.32 for Step 1; Δ*F* = .85 for Step 2; Δ*F* = 1.11 for Step 3.

Neither trait rumination, state rumination, nor mind wandering was a significant predictor variable of average fixation duration (Table 5).

**Table 5:** Predictors of the number of average fixation duration.

	*B*	*SE(b)*	*β*	t	*p*
**Step1**					
(Constant)	1120.59	365.36		3.07	.003
Trait Rumination	-103.16	77.68	-.17	-1.33	.189
**Step2**					
(Constant)	1156.78	363.20		3.19	.002
Trait Rumination	-71.85	80.19	-0.12	-0.90	.374
MW	-83.58	59.50	-0.19	-1.41	.166
**Step3**					
(Constant)	1480.08	440.50		3.36	.001
Trait Rumination	-98.77	82.45	-0.17	-1.20	.236
MW	-98.44	60.29	-0.22	-1.63	.335
State Rumination	-109.39	85.35	-0.18	-1.28	.205

Note: *R*^2^ = .03 for Step 1; Δ*R*^2^ = .03 for Step 2; Δ*R*^2^ = .03 for Step 3.

Note: *F* = 1.76 for Step 1; Δ*F* = 1.97 for Step 2; Δ*F* = 1.64 for Step 3.

## Discussion

Building on previous research [31, 32], we found both trait and state rumination to have a marginally significant effect on TRIs but not on TUTs, that is, on thoughts about the task which actually interfere with performance of the task itself [32]. It is necessary to note that in this study, participants scored highly on trait rumination, leading to a bias in our sample. When using both state and trait rumination to predict TRIs, it may be that one cannot dissociate one from the other, especially not in such a highly ruminative group. Previous studies investigating the relationship between rumination and task-related MW seem to have either only used a trait [32] or a state measure of rumination [31]. In our study, however, we included both measures so that we could assess the success of a validated ruminative state induction procedure and discovered how volatile trait rumination really can be. Importantly, it may be that keeping high trait ruminators in a ruminative state keeps them in that rigid form of thinking. Meanwhile, distracting high trait ruminators from their sticky thoughts enables them to momentarily detach from their rigid patterns of thought [40]. As rumination at time 2 was measured only at the end of the experiment, it is not that surprising that trait rumination scores reduced significantly only in the control group, but that there was no significant interaction between the induction conditions and rumination at times 1 and 2. Any differences found may be a result of a combination between the induction procedures and the word association game. In the control condition, participants were further distracted by the word association game after being asked to think about random thoughts such as “the structure of a long bridge” and “a row of shampoo bottles on display” [31]. In the rumination condition, any increases caused by the rumination induction were likely counteracted by the distracting effects of the word association game. In order to find out if any differences in trait rumination were a result of the manipulation alone, it would have been necessary to measure trait rumination directly after the induction.

As expected, participants in the ruminative state condition generated more negative associations than in the control condition. However, what remains unclear, is why only SWN yielded significant results. This may be due to the fact that it rated words as having positive, objective, and negative scores, while LIWC only rated words for their positivity and negativity. As SWN rated words for these three categories, it seemed to be able to pry apart the objectivity from the positivity and negativity scores of words. In LIWC, on the other hand, a word’s objectivity was likely conflated with either its positivity or negativity scores. Regardless, the finding that ruminative state is generally associated with negative word associations is in line with previous studies that emphasised the effect of rumination not only on negative affect [91, 92], but also on negative cognition [41] and demonstrates the value of analyzing language usage in the exploration of psychological processes. However, when controlling for trait rumination, we did not find any effects of state rumination on the polarity of lexical associations. Indeed, the design of our current study did not include measures for the possible effects of negative mood on negative cognitions. Although experimental studies indicate that rumination increases negative mood relative to distraction, the effect of rumination alone is less clear. In future experiments, it would be interesting to pry apart the effect of rumination, negative mood, and negative cognitions in a more complex experimental design.

The results of our experimental investigation suggest that rumination may play an important role in disrupting the establishment of connection between interaction partners. Previous research concerning pitch and MW yielded mixed findings; Drummond and Litman (2010) [93] found that minimum pitch is a powerful predictor of MW when reading texts aloud. Franklin, Mooneyham, Baird, and Schooler (2014) [94], on the other hand, found no evidence for differences in pitch or pitch variability during MW; however, they did find MW to be was associated with less variability in volume when reading aloud [94]. Rumination, as a type of MW which tends to be fixed on a single theme or topic and is marked by a high degree of automatic constraints [33], attenuates the normal variation in speaker’s pitch in relation to a conversation partner’s pitch. This falls in line with research that has associated rumination with reduced variability in physiological cues, including heart rate variability, which suggests rumination is associated with higher levels of cognitive inflexibility [17, 95–97]. Our study is novel in that it demonstrates that rumination not only predicts reduced variability in physiological cues, it also attenuates pitch imitation which occurs during interactions. Although we framed our experiment in a social, interactive setting, a word association game with an Embodied Conversational Agent only taps into particular aspects of social interactions, and does not take into account their full complexity. Despite this limitation, automatic imitation is one of the most-basic nonverbal components of successful human interactions, and plays an essential role in creating rapport, empathy, and social bonding. In our study, both state and trait rumination were related to a lack of accommodation to the Embodied Conversational Agent’s high and low pitches. This is in line with a previous finding regarding the effect of self-focus –an important feature of rumination– on reduced gesture imitation [62]. While pitch convergence indicates rapport and desire for social approval, pitch divergence may be interpreted as speakers’ desire to be seen as dissimilar and wish to increase the social distance between themselves and their communication partners [50].

Our study demonstrated that trait rumination is related to an increased amount of blinks during an interactive game, which indicates a decoupling of attention from the external environment and a focus on internal thoughts [72]. Smilek and colleagues (2010) [72] found that participants blinked more whenever mind wandering than when focused on reading. Beyond previous findings relating mind wandering as a state to more blinks, our study suggests that rumination, as a stable trait, is also related to more blinks. Although rumination (as both a state and trait) may serve to exacerbate task-related mind wandering, the two cannot be equated, and hence it would be valuable to pry apart actual ruminative MW from from task-related MW episodes during a task. Furthermore, contrary to our predictions that ruminative MW would be related to less saccades, we instead found mind wandering in general to be related to more saccades, while finding no effects for fixation count and fixation duration. It is important to note that the methodology of previous studies upon which we based our predictions differed considerably from ours. Previous studies investigated eye movements in relation to rumination during either an emotional dot-probe task or during abstract animations. With regards to mind wandering, Reichle and colleagues (2010) [67], Smilek and colleagues (2010) [72], Uzzaman and Joordens (2011) [73], and Faber and colleagues (2017) [74], used self-caught or probe-caught measures, to distinguish between periods of MW and focus during reading and only analysed eye movement features from a short period of time preceding each auditory probe. In our study, we used a retrospective measure of MW, and accordingly, measured average eye movement parameters during the entire word association task. Indeed, online thought probes are arguably a more reliable method for measuring mind wandering [33], reducing the probability of confabulation as a result of having to retrospectively assess the content of one’s thoughts over a long period of time [3]. Intermittent thought probes would more accurately pinpoint the moments of time during which participants were mind wandering; however, they would disrupt the natural and automatic flow as well as the covert nature of the word association game.

## Conclusion

In our study, we examined the relationship between rumination and mind wandering and their impact on social cognitive mechanisms that support successful interactions with others. First, we found rumination to marginally predict task-related interferences, suggesting a possible directionality in the relation between rumination and task-related mind wandering. Mind wandering is a broad term, which encompasses a wide variety of self-generated thoughts; hence, rumination elicits a particular type of mind wandering—rigid, self-focused and repetitive—and overlaps with thoughts that are related to a particular task, but interfere with performance of the task itself.

Second, we observed that the emotional valence of lexical associations generated by participants in a condition where a ruminative state was induced was more frequently negative when compared to a control group. Next to that, both trait rumination and a ruminative state led to a decrease in pitch imitation, a more or less automatic process that is used to signal rapport and group membership. It also resulted in an increased number of blinks during the interaction, suggesting that participants were not engaging with their interaction partner. Taken together, these results suggest that ruminative MW may lead to an increased social distance and have the potential of disrupting our social relations. Considering that a substantial portion of our lives is social and interactive, our data highlight the need for further studies of MW in interactive environments.

## Supporting information

S1 File Rumination Inventory. (DOCX)

S2 File Rumination induction. (DOCX)

S3 File Word list. (DOCX)

S4 File Thought component of the Dundee Stress State Questionnaire. (DOCX)

S5 File Relaxing video. (DOCX)

S6 File Rumination and MW data, syntax and output. (ZIP)

S7 File Lexical associations data, syntax and output. (ZIP)

S8 File Pitch data, syntax and output. (ZIP)

S9 File Eye Movement data, syntax and output. (ZIP)

## References

[1] Franklin MS, Mrazek MD, Anderson CL, Smallwood J, Kingstone A, Schooler JW. The silver lining of a mind in the clouds: Interesting musings are associated with positive mood while mind-wandering. Frontiers in Psychology. 2013;4(AUG). 10.3389/fpsyg.2013.00583 PMC3755259 24009599

[2] Killingsworth MA, Gilbert DT. A Wandering Mind Is an Unhappy Mind. Science. 2010;330(6006):932–932. 10.1126/science.1192439 21071660 10.1126/science.1192439

[3] Irving ZC. Mind-wandering is unguided attention: accounting for the “purposeful” wanderer. Philosophical Studies. 2016;173(2):547–571. 10.1007/s11098-015-0506-1

[4] Klinger E. Goal commitments and the content of thoughts and dreams: Basic principles. Frontiers in Psychology. 2013;4(JUL):1–17. 23874312 10.3389/fpsyg.2013.00415 PMC3708449

[5] Smallwood J, Schooler JW. The restless mind. Psychological Bulletin and Review. 2006;132(6):946–958. 10.1037/0033-2909.132.6.946 10.1037/0033-2909.132.6.946 17073528

[6] Smallwood J, Schooler JW. The Science of Mind Wandering: Empirically Navigating the Stream of Consciousness. Annual Review of Psychology. 2015;66(1):487–518. 10.1146/annurev-psych-010814-015331 25293689 10.1146/annurev-psych-010814-015331

[7] Stawarczyk D, Majerus S, Maj M, Van der Linden M, D’Argembeau A. Mind-wandering: Phenomenology and function as assessed with a novel experience sampling method. Acta Psychologica. 2011;136(3):370–381. 10.1016/j.actpsy.2011.01.002 21349473 10.1016/j.actpsy.2011.01.002

[8] Krawietz SA, Tamplin AK, Radvansky GA. Aging and mind wandering during text comprehension. Psychology and Aging. 2012;27(4):951–958. 10.1037/a0028831 22686406 10.1037/a0028831

[9] Smallwood J, McSpadden M, Schooler JW. When attention matters: The curious incident of the wandering mind. Memory & Cognition. 2008;36(6):1144–1150. 10.3758/MC.36.6.1144 18927032 10.3758/MC.36.6.1144

[10] McVay JC, Kane MJ. Conducting the train of thought: Working memory capacity, goal neglect, and mind wandering in an executive-control task. Journal of Experimental Psychology: Learning, Memory, and Cognition. 2009;35(1):196–204. 10.1037/a0014104 19210090 10.1037/a0014104 PMC2750806

[11] Mrazek MD, Smallwood J, Franklin MS, Chin JM, Baird B, Schooler JW. The role of mind-wandering in measurements of general aptitude. Journal of Experimental Psychology: General. 2012;141(4):788–798. 10.1037/a0027968 22468669 10.1037/a0027968

[12] McVay JC, Kane MJ. Drifting from slow to “d’oh!”: Working memory capacity and mind wandering predict extreme reaction times and executive control errors. Journal of Experimental Psychology: Learning, Memory, and Cognition. 2012;38(3):525–549. 10.1037/a0025896 22004270 10.1037/a0025896 PMC3395723

[13] Baird B, Smallwood J, Mrazek MD, Kam JWY, Franklin MS, Schooler JW. Inspired by Distraction. Psychological Science. 2012;23(10):1117–1122. 10.1177/0956797612446024 22941876 10.1177/0956797612446024

[14] Baird B, Smallwood J, Schooler JW. Back to the future: Autobiographical planning and the functionality of mind-wandering. Consciousness and Cognition. 2011;20(4):1604–1611. 10.1016/j.concog.2011.08.007 21917482 10.1016/j.concog.2011.08.007

[15] Mooneyham BW, Schooler JW. The costs and benefits of mind-wandering: A review. Canadian Journal of Experimental Psychology/Revue canadienne de psychologie expérimentale. 2013;67(1):11–18. 10.1037/a0031569 23458547 10.1037/a0031569

[16] Smallwood J, Andrews-Hanna J. Not all minds that wander are lost: The importance of a balanced perspective on the mind-wandering state. Frontiers in Psychology. 2013;4(AUG):1–6. 23966961 10.3389/fpsyg.2013.00441 PMC3744871

[17] Ottaviani C, Shapiro D, Couyoumdjian A. Flexibility as the key for somatic health: From mind wandering to perseverative cognition. Biological Psychology. 2013;94(1):38–43. 10.1016/j.biopsycho.2013.05.003 23680439 10.1016/j.biopsycho.2013.05.003

[18] Tulving E. Multiple memory systems and consciousness. Human neurobiology. 1987;6:67–80. 3305441

[19] Poerio GL, Smallwood J. Daydreaming to navigate the social world: What we know, what we don’t know, and why it matters. Social and Personality Psychology Compass. 2016;10(11):605–618. 10.1111/spc3.12288

[20] Poerio GL, Totterdell P, Miles E. Mind-wandering and negative mood: Does one thing really lead to another? Consciousness and Cognition. 2013;22(4):1412–1421. 10.1016/j.concog.2013.09.012 24149091 10.1016/j.concog.2013.09.012

[21] Watkins E, Mason A. Mood as input and rumination. Personality and Individual Differences. 2002;32(4):577–587. 10.1016/S0191-8869(01)00058-7

[22] McVay JC, Kane MJ. Dispatching the wandering mind? Toward a laboratory method for cuing “spontaneous” off-task thought. Frontiers in Psychology. 2013;4(SEP). 10.3389/fpsyg.2013.00570 24027542 10.3389/fpsyg.2013.00570 PMC3760067

[23] Medea B, Karapanagiotidis T, Konishi M, Ottaviani C, Margulies D, Bernasconi A, et al How do we decide what to do? Resting-state connectivity patterns and components of self-generated thought linked to the development of more concrete personal goals. Experimental Brain Research. 2016; p. 1–13. 10.1007/s00221-016-4729-y PMC6096705 27443852

[24] Smallwood J, Fitzgerald A, Miles LK, Phillips LH. Shifting moods, wandering minds: Negative moods lead the mind to wander. Emotion. 2009;9(2):271–276. 10.1037/a0014855 19348539 10.1037/a0014855

[25] Stawarczyk D, Majerus S, D’Argembeau A. Concern-induced negative affect is associated with the occurrence and content of mind-wandering. Consciousness and Cognition. 2013;22(2):442–448. 10.1016/j.concog.2013.01.012 23466878 10.1016/j.concog.2013.01.012

[26] Marchetti I, Koster EHW, De Raedt R. Mindwandering heightens the accessibility of negative relative to positive thought. Consciousness and Cognition. 2012;21(3):1517–1525. 10.1016/j.concog.2012.05.013 22726693 10.1016/j.concog.2012.05.013

[27] Joormann J. Differential effects of rumination and dysphoria on the inhibition of irrelevant emotional material: Evidence from a negative priming task. Cognitive Therapy and Research. 2006;30(2):149–160. 10.1007/s10608-006-9035-8

[28] Martin LL, Tesser A. Toward a motivational and structural theory of ruminative thought In: Unintended thought. New York: Guilford Press; 1989 p. 306–326.

[29] Ottaviani C, Couyoumdjian A. Pros and cons of a wandering mind: A prospective study. Frontiers in Psychology. 2013;4(AUG):1–9. 23966964 10.3389/fpsyg.2013.00524 PMC3743222

[30] Marchetti I, Koster EHW, Klinger E, Alloy LB. Spontaneous Thought and Vulnerability to Mood Disorders. Clinical Psychological Science. 2016;4(5):835–857. 10.1177/2167702615622383 28785510 10.1177/2167702615622383 PMC5544025

[31] Lyubomirsky S, Kasri F, Zehm K. Dysphoric rumination impairs concentration on academic tasks. Cognitive Therapy and Research. 2003;27(3):309–330. 10.1023/A:1023918517378

[32] Smallwood J, O’Connor RC, Heim D. Rumination, Dysphoria, and Subjective Experience. Imagination, Cognition and Personality. 2005;24(4):355–367. 10.2190/AE18-AD1V-YF7L-EKBX

[33] Christoff K, Irving ZC, Fox KCR, Spreng RN, Andrews-Hanna JR. Mind-wandering as spontaneous thought: a dynamic framework. Nature Reviews Neuroscience. 2016;17(11):718–731. 10.1038/nrn.2016.113 27654862 10.1038/nrn.2016.113

[34] Davis RN, Nolen-Hoeksema S. Cognitive inflexibility among ruminators and nonruminators. Cognitive Therapy and Research. 2000;24(6):699–711. 10.1023/A:1005591412406

[35] Nolen-Hoeksema S, Davis CG. “Thanks for sharing that”: Ruminators and their social support networks. Journal of Personality and Social Psychology. 1999;77(4):801–814. 10.1037/0022-3514.77.4.801 10531672 10.1037//0022-3514.77.4.801

[36] Nolen-Hoeksema S, Morrow J, Fredrickson BL. Response styles and the duration of episodes of depressed mood. Journal of Abnormal Psychology. 1993;102(1):20–28. 10.1037/0021-843X.102.1.20 8436695 10.1037//0021-843x.102.1.20

[37] Hertel PT. Relation between rumination and impaired memory in dysphoric moods. Journal of Abnormal Psychology. 1998;107(1):166–172. 10.1037/0021-843X.107.1.166 9505050 10.1037//0021-843x.107.1.166

[38] Matthews G, Joyner L, Gilliland K, Campbell S, Falconer S, Huggins J. Validation of a comprehensive stress state questionnaire: Towards a state “Big Three?” In: Mervielde I, Deary IJ, De Fruyt F, Ostendorf F, editors. Personality Psychology in Europe. 7th ed Tilburg: Tilburg University Press; 1999 p. 335–350.

[39] Treynor W, Gonzalez R, Nolen-Hoeksema S. Rumination Reconsidered: A Psychometric Analysis. Cognitive Therapy and Research. 2003;27(3):247–259. 10.1023/A:1023910315561

[40] Siegle GJ, Steinhauer SR, Carter CS, Ramel W, Thase ME. Do the Seconds Turn Into Hours? Relationships between Sustained Pupil Dilation in Response to Emotional Information and Self-Reported Rumination. Cognitive Therapy and Research. 2003;27(3):365–382. 10.1023/A:1023974602357

[41] Kuo JR, Edge IG, Ramel W, Edge MD, Drabant EM, Dayton WM, et al Trait Rumination Is Associated with Enhanced Recollection of Negative Words. Cognitive therapy and research. 2012;36(6):722–730. 10.1007/s10608-011-9430-7 25587202 10.1007/s10608-011-9430-7 PMC4289628

[42] Mar RA, Mason MF, Litvack A. How daydreaming relates to life satisfaction, loneliness, and social support: The importance of gender and daydream content. Consciousness and Cognition. 2012;21(1):401–407. 10.1016/j.concog.2011.08.001 22033437 10.1016/j.concog.2011.08.001

[43] Kane MJ, Smeekens BA, von Bastian CC, Lurquin JH, Carruth NP, Miyake A. A combined experimental and individual-differences investigation into mind wandering during a video lecture. Journal of Experimental Psychology: General. 2017;146(11):1649–1674. 10.1037/xge0000362 29094964 10.1037/xge0000362

[44] Wammes J, Seli P, Smilek D. Mind-Wandering in Educational Settings In: Fox KCR, Christoff K, editors. The Oxford Handbook of Spontaneous Thought: Mind Wandering, Creativity, and Dreaming. New York: Oxford University Press; 2018 p. 259–271.

[45] Hutt S, Mills C, White S, Donnelly PJ, D’Mello SK. The Eyes Have It: Gaze-based Detection of Mind Wandering during Learning with an Intelligent Tutoring System. In: Proceedings of the 9th International Conference on Educational Data Mining. International Educational Data Mining Society.; 2016. p. 86–93.

[46] Mills C, D’mello S, Bosch N, Olney AM. Mind Wandering During Learning with an Intelligent Tutoring System. In: International Conference on Artificial Intelligence in Education; 2015. p. 267–276.

[47] Lakin JL, Jefferis VE, Cheng CM, Chartrand TL. The chameleon effect as social glue: Evidence for the evolutionary significance of nonconscious mimicry. Journal of Nonverbal Behavior. 2003;27(3):145–162. 10.1023/A:1025389814290

[48] Rauthmann JF, Seubert CT, Sachse P, Furtner MR. Eyes as windows to the soul: Gazing behavior is related to personality. Journal of Research in Personality. 2012;46(2):147–156. 10.1016/j.jrp.2011.12.010

[49] Mattheij R, Postma-Nilsenová M, Postma E. Mirror mirror on the wall: Is there mimicry in you all? Journal of Ambient Intelligence and Smart Environments. 2015;7(2):121–132.

[50] Postma-Nilsenová M, Brunninkhuis N, Postma E. Eye Gaze Affects Vocal Intonation Mimicry. Cognitive Psychology. 2013; p. 1139–1144.

[51] Giles H. Communication Accommodation Theory: “When in Rome …” or Not! In: Baxte L, Braithwaite DO, editors. Engaging Theories in Interpersonal Communication: Multiple Perspectives. Thousand Oaks: SAGE Publications; 2008 p. 161–174.

[52] Harwood J, Soliz J, Lin MC. Communication Accommodation Theory: An Intergroup Approach to Family Relationships In: Engaging Theories in Family Communication: Multiple Perspectives Engaging theories in family communication: Multiple perspectives. Thousand Oaks: SAGE Publications; 2006 p. 19–34.

[53] Maddux WW, Mullen E, Galinsky AD. Chameleons bake bigger pies and take bigger pieces: Strategic behavioral mimicry facilitates negotiation outcomes. Journal of Experimental Social Psychology. 2008;44(2):461–468. 10.1016/j.jesp.2007.02.003

[54] Pardo JS. On phonetic convergence during conversational interaction. The Journal of the Acoustical Society of America. 2006;119(4):2382–2393. 10.1121/1.2178720 16642851 10.1121/1.2178720

[55] Shockley K, Sabadini L, Fowler CA. Imitation in shadowing words. Perception & psychophysics. 2004;66(3):422–429. 10.3758/BF03194890 15283067 10.3758/bf03194890

[56] Babel M, Bulatov D. The Role of Fundamental Frequency in Phonetic Accommodation. Language and Speech. 2012;55(2):231–248. 10.1177/0023830911417695 22783633 10.1177/0023830911417695

[57] Gorisch J, Wells B, Brown GJ. Pitch Contour Matching and Interactional Alignment across Turns: An Acoustic Investigation. Language and Speech. 2012;55(1):57–76. 10.1177/0023830911428874 22480026 10.1177/0023830911428874

[58] Postma-Nilsenovà M, Postma E. Auditory perception bias in speech imitation. Frontiers in Psychology. 2013;4(NOV). 10.3389/fpsyg.2013.00826 24204361 10.3389/fpsyg.2013.00826 PMC3817513

[59] Nilsenová M, Swerts M. Prosodic Adaptation in Language Learning In: Romero-Trilho J, editor. Pragmatics and Prosody in English Language Teaching. Springer, Dordrecht; 2012 p. 77–94.

[60] Spengler S, Brass M, Kühn S, Schütz-Bosbach S. Minimizing motor mimicry by myself: Self-focus enhances online action-control mechanisms during motor contagion. Consciousness and Cognition. 2010;19(1):98–106. 10.1016/j.concog.2009.12.014 20116291 10.1016/j.concog.2009.12.014

[61] Dijksterhuis a, Bargh J. The perception-behavior expressway: Automatic effects of social perception on social behavior. Advances in experimental social psychology. 2001;33:1–38. 10.1016/S0065-2601(01)80003-4

[62] van Baaren RB, Maddux WW, Chartrand TL, de Bouter C, van Knippenberg A. It takes two to mimic: Behavioral consequences of self-construals. Journal of Personality and Social Psychology. 2003;84(5):1093–1102. 10.1037/0022-3514.84.5.1093 12757151 10.1037/0022-3514.84.5.1093

[63] Gaebel W, Wölwer W. Facial expression and emotional face recognition in schizophrenia and depression. European Archives of Psychiatry and Clinical Neuroscience. 1992;242(1):46–52. 10.1007/BF02190342 1390955 10.1007/BF02190342

[64] Wexler BE, Levenson L, Warrenburg S, Price LH. Decreased perceptual sensitivity to emotion-evoking stimuli in depression. Psychiatry Research. 1994;51(2):127–138. 10.1016/0165-1781(94)90032-9 8022947 10.1016/0165-1781(94)90032-9

[65] Knapp M, Hall J, Horgan T. The effects of eye behavior on human communication In: Yasutake D, Solan C, Badiner J, editors. Nonverbal communication in human interaction. 8th ed Wadsworth: Cengage Learning; 2014 p. 295–322.

[66] Matsumoto K, Shibata S, Seiji S, Mori C, Shioe K. Factors influencing the processing of visual information from non-verbal communications. Psychiatry and Clinical Neurosciences. 2010;64(3):299–308. 10.1111/j.1440-1819.2010.02077.x 20408990 10.1111/j.1440-1819.2010.02077.x

[67] Reichle ED, Reineberg AE, Schooler JW. Eye Movements During Mindless Reading. Psychological Science. 2010;21(9):1300–1310. 10.1177/0956797610378686 20679524 10.1177/0956797610378686

[68] Terburg D, Hooiveld N, Aarts H, Kenemans JL, van Honk J. Eye tracking unconscious face-to-face confrontations: Dominance motives prolong gaze to masked angry faces. Psychological Science. 2011;22(3):314–319. 10.1177/0956797611398492 21303993 10.1177/0956797611398492

[69] Duque A, Sanchez A, Vazquez C. Gaze-fixation and pupil dilation in the processing of emotional faces: The role of rumination. Cognition and Emotion. 2014;28(8):1347–1366. 10.1080/02699931.2014.881327 24479673 10.1080/02699931.2014.881327

[70] Hilt LM, Leitzke BT, Pollak SD. Can’t Take My Eyes Off of You: Eye Tracking Reveals How Ruminating Young Adolescents Get Stuck. Journal of Clinical Child and Adolescent Psychology. 2017;46(6):858–867. 10.1080/15374416.2015.1121824 26909708 10.1080/15374416.2015.1121824 PMC4996756

[71] He J Becic ELYC, McCarley JS. Mind Wandering Behind the Wheel: Performance and Oculomotor Correlates. Human Factors: The Journal of the Human Factors and Ergonomics Society. 2011;53(1):13–21. 10.1177/0018720810391530 10.1177/0018720810391530 21469530

[72] Smilek D, Carriere JSA, Cheyne JA. Out of Mind, Out of Sight: Eye Blinking as Indicator and Embodiment of Mind Wandering. Psychological Science. 2010;21(6):786–789. 10.1177/0956797610368063 20554601 10.1177/0956797610368063

[73] Uzzaman S, Joordens S. The eyes know what you are thinking: Eye movements as an objective measure of mind wandering. Consciousness and Cognition. 2011;20(4):1882–1886. 10.1016/j.concog.2011.09.010 21968150 10.1016/j.concog.2011.09.010

[74] Faber M, Bixler R, D’Mello SK. An automated behavioral measure of mind wandering during computerized reading. Behavior Research Methods. 2017; p. 1–17. 28181186 10.3758/s13428-017-0857-y

[75] Blanchard N, Bixler R, Joyce T, D’Mello S. Automated Physiological-Based Detection of Mind Wandering during Learning In: Trausan-Matu S, Boyer KE, Crosby M, Panourgia K, editors. Intelligent Tutoring Systems. Springer, Cham; 2014 p. 55–60.

[76] Recarte MA, Pérez E, Conchillo A, Nunes LM. Mental workload and visual impairment: differences between pupil, blink, and subjective rating. The Spanish journal of psychology. 2008;11(2):374–385. 18988425

[77] Goldinger SD. Echoes of echoes? An episodic theory of lexical access. Psychological review. 1998;105(2):251–79. 10.1037/0033-295X.105.2.251 9577239 10.1037/0033-295x.105.2.251

[78] Dufour S, Nguyen N. How much imitation is there in a shadowing task? Frontiers in psychology. 2013;4:346. 10.3389/fpsyg.2013.00346 PMC3689145 23801974

[79] Simonsohn U. Small Telescopes: Detectability and the Evaluation of Replication Results. Psychological Science. 2015;26(5):559–569. 10.1177/0956797614567341 25800521 10.1177/0956797614567341

[80] McIntosh WD, Martin LL. The cybernetics of happiness: The relation of goal attainment, rumination, and affect In: Emotion and social behavior. Review of personality and social psychology, Vol. 14 Thousand Oaks: Sage Publications,; 1992 p. 222–246.

[81] Behar E, McGowan SK, McLaughlin KA, Borkovec TD, Goldwin M, Bjorkquist O. Concreteness of Positive, Negative, and Neutral Repetitive Thinking About the Future. Behavior Therapy. 2012;43(2):300–312. 10.1016/j.beth.2011.07.002 22440067 10.1016/j.beth.2011.07.002 PMC4086674

[82] Lyubomirsky S, Nolen-Hoeksema S. Self-perpetuating properties of dysphoric rumination. Journal of Personality and Social Psychology. 1993;65(2):339–349. 10.1037/0022-3514.65.2.339 8366423 10.1037//0022-3514.65.2.339

[83] Boersma P. Praat, a system for doing phonetics by computer. Glot International. 2001;5(9/10):341–347.

[84] Schaafsma J, Krahmer E, Postma M, Swerts M, Balsters M, Vingerhoets A. Comfortably Numb? Nonverbal Reactions to Social Exclusion. Journal of Nonverbal Behavior. 2015;39(1):25–39. 10.1007/s10919-014-0198-9

[85] Hess W. Pitch Determination of Speech Signals vol. 3 of Springer Series in Information Sciences. Berlin: Springer Berlin Heidelberg; 1983.

[86] Leemann A. Swiss German Intonation Patterns vol. 10 of Studies in Language Variation. Amsterdam: John Benjamins Publishing Company; 2012.

[87] Ishi CT, Sakakibara KI, Ishiguro H, Hagita N. A method for automatic detection of vocal fry. IEEE Transactions on Audio, Speech and Language Processing. 2008;16(1):47–56. 10.1109/TASL.2007.910791

[88] Pennebaker JW, Francis ME, Booth RJ. Linguistic Inquiry and Word Count; 2001.

[89] Tausczik YR, Pennebaker JW. The Psychological Meaning of Words: LIWC and Computerized Text Analysis Methods. Journal of Language and Social Psychology. 2010;29(1):24–54. 10.1177/0261927X09351676

[90] Esuli A, Sebastiani F. SENTIWORDNET: A Publicly Available Lexical Resource for Opinion Mining. Proceedings of the 5th Conference on Language Resources and Evaluation. 2006; p. 417–422.

[91] Thomsen DK. The association between rumination and negative affect: A review. Cognition and Emotion. 2006;20(8):1216–1235. 10.1080/02699930500473533

[92] Takano K, Sakamoto S, Tanno Y. Ruminative self-focus in daily life: Associations with daily activities and depressive symptoms. Emotion. 2013;13(4):657–667. 10.1037/a0031867 23527502 10.1037/a0031867

[93] Drummond J, Litman D. In the Zone: Towards Detecting Student Zoning Out Using Supervised Machine Learning In: LNCS. vol. 6095; 2010 p. 306–308.

[94] Franklin MS, Mooneyham BW, Baird B, Schooler JW. Thinking one thing, saying another: The behavioral correlates of mind-wandering while reading aloud. Psychonomic Bulletin & Review. 2014;21(1):205–210. 10.3758/s13423-013-0468-2 23807760 10.3758/s13423-013-0468-2

[95] Williams DP, Feeling NR, Hill LK, Spangler DP, Koenig J, Thayer JF. Resting Heart Rate Variability, Facets of Rumination and Trait Anxiety: Implications for the Perseverative Cognition Hypothesis. Frontiers in human neuroscience. 2017;11:520 10.3389/fnhum.2017.00520 29163100 10.3389/fnhum.2017.00520 PMC5671536

[96] Cropley M, Plans D, Morelli D, Sütterlin S, Inceoglu I, Thomas G, et al The Association between Work-Related Rumination and Heart Rate Variability: A Field Study. Frontiers in Human Neuroscience. 2017;11:27 10.3389/fnhum.2017.00027 28197087 10.3389/fnhum.2017.00027 PMC5281594

[97] Woody ML, McGeary JE, Gibb BE. Brooding rumination and heart rate variability in women at high and low risk for depression: Group differences and moderation by COMT genotype. Journal of Abnormal Psychology. 2014;123(1):61–67. 10.1037/a0035450 24661160 10.1037/a0035450 PMC4107362

