# Supplementary material for: Ruminative minds, wandering minds: Effects of rumination and mind wandering on lexical associations, pitch imitation and eye behaviour
Source: PLoS One. 2018 Nov 19;13(11):e0207578. doi: 10.1371/journal.pone.0207578 (PMC6242373; doi:10.1371/journal.pone.0207578)
Supplement: S1 File — (DOCX) [file pone.0207578.s001.docx]

**S1 File. Measure of Rumination.**

*Rumination Inventory*

DIRECTIONS: Indicate how well each of the statements below describes you by filling in the appropriate number.

1. I seldom think about things that happened in the past.

Does not A/1 : B/2 : C/3 : D/4 : E/5 : F/6 : G/7 Describes

Describe Me Well

Me Well

2. I often get distracted from what I am doing with thoughts about something else.

Does not A/1 : B/2 : C/3 : D/4 : E/5 : F/6 : G/7 Describes

Describe Me Well

Me Well

3. If I don’t want to think about something, I’m able to just stop thinking about it.

Does not A/1 : B/2 : C/3 : D/4 : E/5 : F/6 : G/7 Describes

Describe Me Well

Me Well

4. I often think about what my life will be like in the future.

Does not A/1 : B/2 : C/3 : D/4 : E/5 : F/6 : G/7 Describes

Describe Me Well

Me Well

5. When I have a problem, I tend to think about it a lot of the time.

Does not A/1 : B/2 : C/3 : D/4 : E/5 : F/6 : G/7 Describes

Describe Me Well

Me Well

6. I often become “lost in thought”.

Does not A/1 : B/2 : C/3 : D/4 : E/5 : F/6 : G/7 Describes

Describe Me Well

Me Well

7. When I know that I am going to have an important talk or an argument with someone in the near future, I rehearse in my mind what I will say and what they will probably say in response.

Does not A/1 : B/2 : C/3 : D/4 : E/5 : F/6 : G/7 Describes

Describe Me Well

Me Well

8. Sometimes I feel like I have no control over my thoughts.

Does not A/1 : B/2 : C/3 : D/4 : E/5 : F/6 : G/7 Describes

Describe Me Well

Me Well

9. I have no trouble focusing all of my attention on one thing.

Does not A/1 : B/2 : C/3 : D/4 : E/5 : F/6 : G/7 Describes

Describe Me Well

Me Well

10. When I don’t understand something that happens, I tend to run it over in my mind until I can make sense out of it.

Does not A/1 : B/2 : C/3 : D/4 : E/5 : F/6 : G/7 Describes

Describe Me Well

Me Well

**NOTE:** We asked participants to fill out this questionnaire online, using www.qualtrics.com. Items 1, 3, and 9 had to be reverse coded.
