# Supplementary material for: Ruminative minds, wandering minds: Effects of rumination and mind wandering on lexical associations, pitch imitation and eye behaviour
Source: PLoS One. 2018 Nov 19;13(11):e0207578. doi: 10.1371/journal.pone.0207578 (PMC6242373; doi:10.1371/journal.pone.0207578)
Supplement: S2 File — (DOCX) [file pone.0207578.s002.docx]

**S2 File. Rumination Manipulation.**

The Rumination Induction Procedure was used as a manipulation in this study. Half of the participants were in the Rumination Induction condition ( coded as condition 1), and the other half were in the control condition (coded as condition 2).

*Experimental Condition: Rumination Induction*

Packet A (Rumination)

***********************

Rumination Induction

PACKET A: THINKING-OUT-LOUD (TIMED TASK)

(TIMED TASK)

Instructions:

For the next few minutes, try your best to focus your attention on each of the ideas on the following pages.

Read each item slowly and silently to yourself. As you read the items, use your imagination and concentration to focus your mind on each of the ideas. Spend a few moments visualizing and concentrating on each item.

Please continue until the experimenter returns.

Think about:

1. the physical sensations you feel in your body
2. your character and who you strive to be
3. the degree of clarity in your thinking right now
4. why you react the way you do
5. the way you feel inside
6. the possible consequences of your current mental state
7. how similar/different you are relative to other people
8. what it would be like if your present feelings lasted
9. why things turn out the way they do
10. trying to understand your feelings
11. how awake/tired you feel now
12. the amount of tension in your muscles
13. whether you are fulfilled
14. your physical appearance
15. whether you feel stressed right now
16. the long-term goals you have set
17. the amount of certainty you feel
18. your present feelings of fatigue/energy
19. possible explanations for your physical sensations
20. how hopeful/hopeless you are feeling
21. the level of motivation you feel right now
22. the degree of helplessness you feel
23. the degree of calmness/restlessness you feel
24. the possible consequences of the way you feel
25. what your feelings might mean
26. how sad/happy you are feeling
27. the expectations your family has for you
28. why your body feels this way
29. why you get this way sometimes
30. how passive/active you feel
31. what people notice about your personality
32. the kind of student you are and wish you were
33. how weak/strong your body feels now
34. the degree of relaxation/agitation you feel
35. the kind of person you think you should be
36. the degree of control you feel right now
37. what would happen if your current physical state lasted
38. sitting down and analyzing your personality
39. why you turned out this way
40. the things that are most important in your life
41. how quick/slow your thinking is right now
42. the degree of decisiveness you feel
43. trying to understand who you are
44. how you feel about your friendships
45. whether you have accomplished a lot so far

*Control Condition: Distraction Induction*

Packet B (Distraction)

**************************

PACKET B: IMAGINING IDEAS (TIMED TASK)

Instructions:

For the next few minutes, try your best to focus your attention on each of the ideas on the following pages.

Read each item slowly and silently to yourself. As you read the items, use your imagination and concentration to focus your mind on each of the ideas. Spend a few moments visualizing and concentrating on each item.

Please continue until the experimenter returns.

1. and imagine a boat slowly crossing the Atlantic
2. the layout of a typical classroom
3. the shape of a large black umbrella
4. the movement of an electric fan on a warm day
5. raindrops sliding down a windowpane
6. a double-decker bus driving down a street
7. and picture a full moon on a clear night
8. clouds forming in the sky
9. the layout of the local shopping center
10. and imagine a plane flying overhead
11. fire darting around a log in a fire-place
12. and concentrate on the expression on the face of the Mona Lisa
13. a parking lot at a drive-in
14. two birds sitting on a tree branch
15. the shadow of a stop sign
16. the layout of the local post office
17. the structure of a high-rise office building
18. and picture the Eiffel Tower
19. and imagine a truckload of watermelons
20. the pattern on an Oriental rug
21. the “man in the moon”
22. the shape of the continent of Africa
23. a band playing outside
24. a group of polar bears fishing in a stream
25. the shape of the torch on the Statue of Liberty
26. the shape of the state of California
27. the way the Grand Canyon looks at sunset
28. the structure of a long bridge
29. a train stopped at a station
30. a lone cactus in the desert
31. the shape of the country of Italy
32. a row of shampoo bottles on display
33. a gas station on the side of a highway
34. the fuzz on the shell of a coconut
35. the Presidents' faces on Mount Rushmore
36. and picture the UCR watch tower
37. a band playing "The Star Spangled Banner"
38. the shape of a cello
39. a puddle in the middle of a sidewalk
40. the shape of the United States
41. the baggage claim area at the airport
42. the size of the Statue of Liberty
43. the shape of a baseball glove
44. a freshly painted door
45. the shiny surface of a trumpet

**Note**: The rumination and distraction induction items (e.g., Think about…) should be printed on separate pages. We created a power point presentation and asked participants to go through the items.
