# Supplementary material for: Ruminative minds, wandering minds: Effects of rumination and mind wandering on lexical associations, pitch imitation and eye behaviour
Source: PLoS One. 2018 Nov 19;13(11):e0207578. doi: 10.1371/journal.pone.0207578 (PMC6242373; doi:10.1371/journal.pone.0207578)
Supplement: S3 File — (DOCX) [file pone.0207578.s003.docx]

**S3 File. List of words.**

*List of words uttered by the Embodied Conversational Agent in the word association game:*

add

class

drop

teach

effort

essay

test

exam

fail

goal

grade

group

time

mark

notes

pass

point

quiz

rules

school

work

write/right

**Note:**  The stimuli were uttered in a random order for each participant.

**High and Low Pitch Manipulations:**

High: add, class, effort, exam, goal, grade, time, mark, quiz, work

Low: teach, essay, test, fail, group, notes, pass, rules, school, write
