# Supplementary material for: Ruminative minds, wandering minds: Effects of rumination and mind wandering on lexical associations, pitch imitation and eye behaviour
Source: PLoS One. 2018 Nov 19;13(11):e0207578. doi: 10.1371/journal.pone.0207578 (PMC6242373; doi:10.1371/journal.pone.0207578)
Supplement: S4 File — (DOCX) [file pone.0207578.s004.docx]

**S4 File. Measure of Mind Wandering.**

For the Thinking Component of the Dundee Stress State questionnaire, questions were coded as TUT1- TUT16. Questions 1-8 refer to Task Related Interferences (TRIs), and Questions 9-16 refer to Task Unrelated Thoughts (TUTs)

*Thinking component of the Dundee Stress Questionnaire*

The following set of questions concerns the kinds of thoughts that go through people’s heads during a task. Please indicate how often you had each thought during the test.

**1 – Never 2 – Once 3 – A few times 4 – Often 5 – Very Often**

1. I thought about how I should work more carefully. 1 2 3 4 5
2. I thought about how much time I had left. 1 2 3 4 5
3. I thought about how others have done on the task. 1 2 3 4 5
4. I thought about the difficulty of the task. 1 2 3 4 5
5. I thought about my level of ability. 1 2 3 4 5
6. I thought about the purpose of the experiment. 1 2 3 4 5
7. I thought about how I would feel if I were told how I performed. 1 2 3 4 5
8. I thought about how often I get confused. 1 2 3 4 5
9. I thought about members of my family. 1 2 3 4 5
10. I thought about something that made me feel guilty. 1 2 3 4 5
11. I thought about personal worries. 1 2 3 4 5
12. I thought about something that made me feel angry. 1 2 3 4 5
13. I thought about something that happened earlier today. 1 2 3 4 5
14. I thought about something that happened to me days ago. 1 2 3 4 5
15. I thought about something that happened in the distant past. 1 2 3 4 5
16. I thought about something that might happen in the future. 1 2 3 4 5

**Note:** We asked participants to fill out this questionnaire online with www.qualtrics.com.
