# Supplementary material for: Ruminative minds, wandering minds: Effects of rumination and mind wandering on lexical associations, pitch imitation and eye behaviour
Source: PLoS One. 2018 Nov 19;13(11):e0207578. doi: 10.1371/journal.pone.0207578 (PMC6242373; doi:10.1371/journal.pone.0207578)
Supplement: S5 File — (DOCX) [file pone.0207578.s005.docx]

**S5 File. Aquarium 2hr relax music.**

The video was retrieved from youtube: <http://www.youtube.com/watch?v=VIrBecB746c>

For Demographics: females were coded as 0, and males as 1. Information was also collected about nationality and age.

The rumination inventory was administered twice at T1 (coded as RIQ1-RIQ10) and T2 (coded as RI2Q1-RI2Q10), as a manipulation check.
